# Sources of Health care providers’ Self-efficacy to deliver Health Education: a qualitative study

**DOI:** 10.1186/s12909-018-1448-z

**Published:** 2019-01-09

**Authors:** Fereshteh Zamani-Alavijeh, Marzieh Araban, Tayebeh Fasihi Harandy, Fatemeh Bastami, Mohammad Almasian

**Affiliations:** 1 Department of Health Education and Promotion, School of Health, Isfahan University of Medical Sciences, Isfahan, Iran; 2 Social Determinants of Health Research Centre, Department of Health Education and Promotion, Public Health School, Ahvaz Jundishapur University of Medical Sciences, Ahvaz, Iran; 3 Social Determinants of Health Research Centre, Alborz University of Medical Sciences, Karaj, Iran; 4 Department of the English Language, Faculty of Medicine, Lorestan University of Medical Sciences, Lorestan, Iran

**Keywords:** Self-efficacy, Iranian health educators, Qualitative study

## Abstract

**Background:**

The self-efficacy of educators plays a crucial role in their professional competence and subsequent provision of care. The present study aims to explain the main sources contributing to the development of self-efficacy beliefs among healthcare providers in delivering health education.

**Methods:**

This qualitative study was conducted from 2015 to 2016 in various settings of Isfahan such as hospitals, doctor’s offices, and healthcare centers. Twenty three health educators with an average of 10-year work experience in healthcare participated in the study. Data were collected using semi-structured in-depth individual interviews and were analyzed through conventional content analysis. Prolonged engagement with the participants, maximum variation in the participants’ characteristics, sampling, and member check were among the factors enriching the research.

**Results:**

The six main categories extracted during data analysis included: 1) Quantity and quality of their experience; 2) Encountering unexpected events; 3) Client trust; 4) Self-concept; 5) Professional knowledge and skill; 6) Vicarious experiences.

**Conclusions:**

The study results show two new findings, including “encountering unexpected events” and “client trust”, affecting professional self-efficacy beliefs among healthcare providers in the delivery of health education. The other main findings were extremely similar to Bandura’s theory. These results can be used as a basis in planning and implementing health development educational models for human resources.

## Background

Through designing learning experiences, health education helps to increase the knowledge or influence the attitudes of individuals and communities in order to improve their health [1]. It encourages them to perform appropriate behaviors and helps them to overcome diseases and maintain good health [2]. Studies indicate that health education is an important factor in enhancing client satisfaction, independence, and participation in healthcare programs and promoting healthy behaviors, leading to helpful outcomes such as improved quality of life and better mental status by reducing diseases complications and decreasing anxiety [3–5]. In addition, access to complete information about their own health, disease, and even treatment [2, 6] can be classified as one of the most important rights of clients. Therefore, depriving them of such education and training is unethical [7, 8].

As some of the most important elements of health promotion, health care providers, particularly nurses and family health experts, can play a significant role in health education owing to their greater access to individuals and families and the considerable time they spend with them to take care of them. As a result, they have numerous opportunities to educate them [9, 10]. However, in most cases, they do not properly utilize the methods and principles of health education [2, 10, 11], and some studies even demonstrate that their weak performance leads to acquisition of incorrect information and beliefs by the clients [12, 13].

The results of previous studies have shown that excessive workload and insufficient force, inadequate qualifications of health educators, failure to integrate patient education into the rehabilitation process, lack of job satisfaction, regarding education as unimportant, education by non-professionals, ignoring patients’ rights in education, inappropriate communication skills, physician-oriented atmosphere, conflict and lack of coherence in education, lack of motivation, lack of a rewarding system in the organization, and poor supervision and control, problems in planning the time and space for the program, nurses’ belief in not considering patient education as their duties, insufficient facilities in hospitals and lack of enough time are the most important causes of insufficiency of client education [14–18]. This behavior of healthcare providers, insufficient health education, may be partly attributed to the above factors. However, other influential factors such as self-efficacy probably contribute this behavior. Based on the literature review, it can be mentioned that self-efficacy, as an adaptable factor, can be the strongest predictor of many behaviors. For this reason, we have addressed it in this article [19–23].

Some studies have indicated that educators’ self-efficacy (i.e. their perception of their abilities) plays an important role in the performance of their educational and training duties [24, 25]. In other words, individuals’ anticipations and expectations depend on their judgments about their ability to perform a particular behavior in a specific situation. Therefore, individuals with higher self-efficacy tend to visualize positive outcomes [19, 26, 27]. However, researchers have primarily investigated this factor in clients, and few researchers have examined health educators’ self-efficacy. The results show that if educators believe in their own ability to communicate, work with media, and hold educational programs, their performance will be more successful and satisfactory [28–30]. Further studies are needed to identify this type of self-efficacy and the factors contributing to its formation. An extensive study of the literature and previous studies in Iran has demonstrated that this issue has not been adequately studied in its real context via the real experiences of healthcare providers without presuppositions. As a result, adequate knowledge has not been obtained regarding the factors affecting healthcare providers’ self-efficacy in performing health education. Therefore, the question leading to the present study is that which factors contribute to formation of self-efficacy among healthcare providers in offering health education. Since self-efficacy is a complex issue and is associated with different social and psychological factors, the most appropriate approach to a better understanding of the concept seems to be qualitative methods [31]. It is necessary to conduct qualitative studies to explain and describe the essence of the notions and concepts and their interrelationships in the natural setting of the occurrence of the phenomena [32]. Therefore, this paper aims to explain the main sources contributing to the development of self-efficacy beliefs among healthcare providers in delivering health education.

## Methods

### Research design

This qualitative study, which was conducted using conventional content analysis methods, considers the reality based on the context, and investigates various structures of the phenomenon under study [31, 33].

### Participants and research context

This qualitative study was conducted on 23 health educators from 2015 to 2016 in various settings of Isfahan’s hospitals, doctor’s offices and healthcare centers. The participants included 3 nurses, 6 individuals with M.Sc. degrees in health education, 11 public health workers, 2 social pathologists (i.e. individuals offering counseling services to clients in centers for behavioral disorders.), and 1 individual with a Ph.D. degree in health education and promotion (19 women and 4 men in total) who on average had 10-year work experience in healthcare. At the beginning of each interview, the willingness and ability of the participant to transfer experiences were examined. Purposive sampling was used: Those who had at least 3 years of health education practice were included in the study. Health education services that offered care to the general public were selected as the study settings.

### Ethical considerations

The Ethics Committee of Isfahan University of Medical Sciences approved the study (No.IR.MUI.REC.1395.1.O95). The necessary explanations were presented to the participants concerning the significance, objectives, methodology, the consent process, and the maintenance of confidentiality at all stages of the study. In addition, the participants were informed about the characteristics of the study team and how the results were achieved. The decision on determining the time and place of the interviews was made with the participants’ consent. In a qualitative study, obtaining informed consent from the participants for their voluntary participation in the study is a process; thus, in the whole period of data collection, this issue should be considered and checked. In the current study, the participants’ consent was continuously verified. Although the initial consent forms were obtained at the beginning of the interviews, the participants’ were asked about their consent and convenience to participate in the study during the study. The participants were also assured that they could freely leave the study at any stage.

### Data collection and analysis

Data collection was conducted using semi-structured, in-depth individual interviews. The participants were interested in precisely and clearly explaining and expressing their experiences to the researcher; the data were collected by recording the participants’ voices, but recording interviews seemed to raise concerns in the participants and most participants seemed to be unwilling to talk while their interviews were being recorded. In this regard, they were assured of the confidentiality of their data, and the researchers pledged to delete the recordings after transcribing the interviews. This issue facilitated communication with health educators and gained their trust, resulting in obtaining enriched data and experiencing a more efficient data collection process. Open-ended and general questions were initially employed in the interviews. The participants were requested to freely talk about their experiences as health educators. The interviews began with a general question concerning barriers and facilitators to health education. Afterward, the interviews were continued by asking exploratory questions like “Please explain how you held that session?” The time of each interview varied from 20 to 60 min, depending on the participants’ views, and the interview situation and process. The researcher continued the interviews up to the point where no new data could be obtained. After conducting and recording each interview, it was fully transcribed verbatim. Data analysis was concurrently conducted with data collection using the qualitative method of conventional content analysis in three phases: preparation, organization and reporting.

All interviews were analyzed. In preparing the interviews, a complete interview, which could be regarded as a meaningful unit, was chosen as the most suitable analysis unit. Each interview was reviewed several times to achieve data immersion. To organize the data, open-coding was utilized. Then, the coded data were recorded in coding sheets for further reference, and grouping was carried out after several interviews. By repeating the mentioned process for each new interview, some topics were added until the final pattern emerged. Merging and comparing groups reduced the number of categories. Sub-categories were formed based on similar characteristics, and the category names showed their contents [34].

### Rigor

Spending sufficient time to communicate and collect data and prolonged engagement helped to achieve in-depth data collection and build trust and rapport with the participants. Maximum-variation sampling was employed based on the health educators’ age, gender, job, number of years of work experience and place of residence. To insure that the analysis reflected the participants’ experiences accurately, member check was carried out with several participants, and a number of changes were applied to the data based on this procedure [35].

## Results

Based on the obtained results, the main sources of self-efficacy beliefs in health educators could be divided into 6 categories, including: 1) **Quantity and quality** of their experience; 2) Encountering unexpected events causing self-efficacy reduction; 3) Vicarious experiences; 4) Self-concept; 5) Self-efficacy as a reciprocally interacting influence on the client’s perception of trust; 6) Professional knowledge and skill as a factor enhancing self-efficacy.

### Quantity and quality of their experience

#### Quantity of experience: Lack of experience leading to low Self-efficacy

The study results showed that inexperienced educators initially had low self-efficacy regarding the holding of educational sessions. A participant told the researcher about his first experience with educating others as follows:

“*Because this was my first work experience, I was anxious and worried all night long, as I might not have been able to organize the session well, speak well, or be unable to answer some questions.”*

: “… *When I started the educational session, I felt I was hot and even flushed, and everyone knew I was inexperienced,* (Public health worker, 6 years of work experience).”

#### Quality of experience

##### Individual impression of the quality of the educational effort (previous success as a factor contributing to self-efficacy)

The findings of the study showed that if the educational programs were successfully performed, and relevant successful and satisfactory experiences were obtained, self-efficacy would increase. This increase in self-efficacy occurred not only in future programs, but also in the same session. As one of the participants said: *“This experience helped me understand that I have the ability to educate others. Because before the educational session, I did not have that much belief in my own ability…But at the end of the session, my self-confidence increased, and I understood that if an educational program was delegated to me, I would be able to deal with it.*” (Public health worker, 5 years of work experience).

##### Receiving feedback

A participant expressed his experience of oral feedback received from the clients as follows: *“When addicts who quit see me, they express their gratitude by saying that whatever we have achieved was due to you. This helps me believe in my ability to offer education and counseling to these people.”* (Social pathologist, 11 years of work experience).

A participant who worked in substance abuse treatment centers described his/her experience of observing changes in the target group: “*I have educated and advised drug addicts for four years now. I am proud of myself because I can perform my duty well. Because I see previously-referred individuals who are now healthy and drug-free as a result of training and counseling.”* (Social pathologist, 9 years of work experience).

Another factor affecting the enhancement of self-efficacy among health educators is official feedback:

“*When your boss gives you a low evaluation score, this does not affect your salary, and only your morale suffers, and you think that you do not want to educate people any longer.”* (Public health worker, 10 years of work experience).

*“Each month, I recommend 10 health educators who are active and work well for a pay raise, because I have noticed that this is really effective and helps them believe in their own capabilities.”* (The city health education planner, 10 years of work experience).

### Self-efficacy reduction when encountering unexpected events

#### Unexpectedly large number of clients

Based on the experiences of educators, sometimes they faced unexpected events such as a large number of clients, which reduced their self-efficacy in conducting the health education program. For example, a participant who was invited to hold an educational session at the cultural center of the municipality said: *“In the morning, when I went to conduct the educational program, I thought the audience consisted of 20-30 individuals. However, 150 individuals were present. Because of stress, I became confused. I had not thought there would be so many attendants. I felt I couldn’t handle it. At first, I was nervous….”*)Public health worker, 4 years of work experience).

#### Clients with an unexpected gender composition

Additionally, if it is unexpected by the educator, the clients’ gender can affect the educator’s self-efficacy*. “... especially half of those present were men (I didn’t expect men to participate). It took me a moment to reclaim my self-confidence. I was sure that I could work with the women, but not men … I became anxious ….”* (Public health worker, 4 years of work experience). This participant added, *“Men asked me more questions and also acknowledged and praised me more.”*

There is no common view in this regard, and the experience is different for educators with different backgrounds, such as work experience and the culture of the health educator’s place of residence. A female health educator said “Men show more pride *and you need to pay more attention to the tone of the word so that they don’t feel affronted* ..... How you communicate is important to them*. Particularly illiterate or poorly educated men do not want to learn from female health educators*.” She said “*Older men interact better and ask for training but young men are rebellious and ridicule everything* ... If the nurse is unable to control the situation, she cannot provide effective education *.*... *We often send male nurses to male patients because they are more accepted by male patients*” (Health education supervisor, 15 years of work experience).

### Encountering unfamiliar clients

Inadequate knowledge of the clients, and their characteristics can reduce educators’ self-efficacy. One of the participants said about holding a session in a public hall, *“[Because I didn’t know them] it took me a while to feel normal again. While the audiovisual equipment operator was preparing, the slides for display, with a quick evaluation, I learned about the class personality types and the educational levels of the audience. And I was able to hold the educational session based on this and I gradually felt normal again.”* (Public health worker, 7 years work experience).

### Vicarious experiences

Spending time with other colleagues in the health and treatment system with the same health education context can positively affect health educators’ self-efficacy, and when individuals possess limited previous experience, they are more sensitive to this issue. A participant who was the health educator of a province explained his/her vicarious experience in relation to other colleagues working in the field of health education: *“Once in the spring, my colleague and I went to visit an agricultural field. There were 7 or 8 women farmers there. After greetings, my colleague taught them contraception methods for one hour. He taught them so well that the next day some of them referred to the health center for the TL operation. As a result of this experience, I understood that I could be an effective educator too…*” (The head of the health education affairs of the city, 11 years of work experience).

A participant described the experience of his M.Sc. studies regarding the effect of vicarious experience on self-efficacy, “*For example, when I go somewhere for giving a lecture, while I am waiting for the earlier speaker to finish his/her speech, I get anxious especially in cases where the speaker is not successful, or on the contrary, when he/she has an excellent lecture. Also, I may think that my topic is not as interesting as his/her topic, and, I become nervous whether I can give a good lecture or not.”* (Public health worker, 14 years of work experience).

### Educators’ Self-concept as a source of Self-efficacy beliefs

Participants’ feelings, emotions, ideas, mentality, and images play an important role in understanding their abilities in health education. A participant explained that *“I behave in a manner that children admire me, and admit that I am an authority or a role model to be followed on this issue. Because I feel that they are searching for a role model at this age.”* (School Health Nurse, Bachelor of Nursing, 3 years of work experience).

Another participant said that *“I am relaxed in contact with others. I have good interpersonal skills. I am popular among children, so I can influence and educate them and give them advice.”* (School Health Nurse, Bachelor of Nursing, 10 years of work experience).

The results of this study indicate that considering yourself creative is an enhancing factor for self-efficacy. For example, a participant said: “*My instructional creativity has resulted in having an educational program with an effectiveness of 70% and has led to client satisfaction.”* (Nursing health education manager, 13 years of work experience).

Another one expressed her self-concept beliefs by the following statement: “*The clients admire me more. Because they expect a midwife to be tall and have wide shoulders, they assume me more suitable for this job. Since I am married, I can educate sex and midwifery issues better. People do not accept my colleague who is single and has a small body well*.” (Midwife, 10 years of work experience).

One of the participants described his/her self-concept: “*I don’t talk much about a healthy lifestyle (nutrition and exercise) because I feel I am not a good role model (I am overweight). I think this belief about myself can lead to anxiety and feelings of helplessness in every educator.*” (Ph.D. in health education and promotion, Faculty member, 14 years of work experience).

### Self-efficacy as reciprocally interacting influence on the client’s perception of trust

The self-efficacy of the educator is largely affected by clients’ trust and assurance such that if he/she believes that the clients trust him/her, his/her self-efficacy will increase. A participant who worked in a doctor’s office stated that: *“Here, trust is very important, and the client should trust the educator, especially in our field, that is, midwifery and sexual issues. If the clients don’t have confidence in us, we can’t…When the trust and confidence is formed, the counseling could be offered well.”* (Midwife, 13 years of work experience).

Another participant who had held an educational session for the caregivers of Alzheimer’s patients’ said, “*I started to speak. I tried to earn their trust by telling them that I have the same problem and I take care of my mother who has the same problem. I was aware of the details of the disease clinically and in terms of the awareness and attitudes of the family members regarding the disease.”* (Public health worker, 4 years of work experience).

Not only can earning the trust of the clients increase health educators’ self-efficacy, but also their self-efficacy can help to earn the clients’ trust and confidence. A participant said, *“We should be able to communicate, not only with words, but also with the type of eye contact we make, so that we can earn their trust, and educate and offer them counseling.”* (Midwife, 13 years of work experience).

One of the participants described his/her experience in earning the trust of the clients and its effects on his/her self-efficacy thus: *“Sometimes, when I am invited to educate healthcare personnel, if they do not trust me and if I feel that I can’t teach acceptable issues, I become nervous and anxious. First, I try to earn their trust…For example, there were clients in my class that I felt that did not feel any need for what I taught. They thought my speech was not relevant to them, so they did not trust me adequately. I conducted a research study in this regard, and for the next session, I started my educational session by presenting a review study in my chosen field which was conducted by one of the prominent people in their field. Thus they understood that some people who outshone in their field used these methods, and in this way, their trust and confidence increased, and I succeeded.”* (Ph.D. in health education and promotion, Faculty member, 14 years of work experience).

### Professional knowledge and skill as a factor enhancing Self-efficacy

Professional knowledge and skill are one of the factors enhancing educators’ self-efficacy. A participant said, *“I am familiar with the PEN-3 model and its use, I can successfully educate women about the pap-smear test.”* (Midwife, health educator, 5 years of work experience). Another participant said, *“I am familiar with the practical skills for health education. For instance, I can describe the self-care program very tangibly. For example, if you want to go walking, when do you have the time, and how do you like to do it.”* (Nurse, 12 years of work experience).

A participant suggests that lack of knowledge about up-to-date information is a factor reducing professional self-efficacy: “...*In that session, the clients talked about issues that made me feel that they were more up-to-date than me. That is because they were constantly in contact with their doctors, and the things that I knew were fossilized in the books, and that is why I could not educate them well.”* (Public health worker, 13 years of work experience).

A participant pointed out that, *“For example, I go to a school to teach children a fertility health program. The first thing I need to know is needs assessment to learn what necessities they have, at what level their awareness is on this specific topic, what needs they have in mind, so that I can conduct a good educational program …”* (midwife, 13 years of work experience).

Another one described the effect of professional knowledge and skill like this: *“If at the time of the educational program you are constantly worried that you have not mastered what you are teaching, then you will be stressed and will feel that you can’t control the class.”* (Master of Science in Nursing, 10 years of work experience).

## Discussion

The present study aimed to explain the factors contributing to the formation of health educational self-efficacy among health care providers. The results indicated existence of a new classification with 6 categories of factors affecting health educational self-efficacy. Based on the participants’ statements, self-efficacy means the educator’s faith in his/her own abilities to hold educational programs and sessions, which were considered an important factor in their success and in performing their duties. In one study conducted by Haghbaghery and Salsali [36], the participants also emphasized the key role of self-efficacy in creating professional feelings and using its power. Factors affecting self-efficacy as extracted by this study are largely consistent with the sources proposed by Bandura [19, 37, 38], but there were some differences. In the following, each of these factors is compared to the sources proposed by Bandura and the results obtained from other studies.

The first factor presented in this study was “the quantity and quality of their experience”. In previous studies [19, 37, 38], only the “previous experience factor” is mentioned. However, the present study redefined and explained the educators’ experiences in both quantitative and qualitative dimensions, such that being inexperienced was assumed as the quantitative dimension, and personal interpretation and impression regarding the quality of previous experiences of educating others were also assumed as a qualitative dimension (referring to prior individual successes), which were both presented as the factors affecting self-efficacy. These two concepts are in line with the first source of self-efficacy proposed by Bandura. Based on Bandura’s theory, successful experience in the performance of some behaviors can enhance self-efficacy, while experiencing a failure will lead to weakened self-efficacy [19, 37, 38]. Furthermore, the result of a review study by Usher indicated that the most important sources of self-efficacy beliefs in school students were mastery experiences [39]. However, these studies do not report anything about “lack of previous experience” as a factor affecting self-efficacy.

Additionally, the present study showed that, in addition to individual perceptions of failure and success, other people’s perceptions, especially those of officials and clients, play an important role in increasing self-efficacy. According to Bandura’s theory and the results obtained by the present study and other studies [19, 40], it can be inferred that social encouragement is another way of increasing self-efficacy. People might obtain an incorrect understanding of their own competence by incorrectly evaluating their own knowledge, abilities, and skills. The results of the present study indicate that feedback from clients and officials plays a crucial role in reemphasizing the abilities of health educators and their own competence interpretation. This finding is similar to that of the study by Usher [39]. This review study reports social and oral encouragements as the third source of self-efficacy according to Bandura. In this regard, students not skilled in self-evaluation depend on the feedback and others’ evaluative judgments about their own educational performance. These supportive messages boost students’ efforts and self-confidence to attempt to succeed. Similar to previous studies [41, 42], the present study showed that receiving supportive feedback from the environment enhanced individuals’ self-efficacy.

The second factor affecting self-efficacy was “encountering unexpected events”. This factor was not reported in previous studies and was not directly in line with the sources proposed by Bandura [19, 26, 27]. In the present study, facing events such as clients’ unexpected gender composition, unexpectedly large number of clients, and unfamiliarity with them, particularly among inexperienced educators, reduced their self-efficacy due to the participants’ stress and nervousness in encountering the above-mentioned situations. In this respect, an equivalent for the concept “the ability to encounter unexpected events” can be found in previous studies as “resiliency”. However, given that resiliency is defined as skills, characteristics, and abilities enabling individuals to adapt to difficulties and challenges [43], it has some differences with the concept extracted in the present study, i.e. “encountering unexpected events”. The latter concept refers to an environmental factor affecting the individual, that is, events that are unpredictable and affect the educators’ self-efficacy. Resiliency is an internal characteristic in individuals. Another difference is that “unexpected events” affect self-efficacy, while resiliency is affected by such events. The results of the present study showed that unexpected events influenced individuals’ self-efficacy in conducting the educational program. However, self-efficacy itself can enhance resiliency and the ability to encounter unexpected events [44]. Certainly, studies have indicated that individuals with lower resiliency are more likely to suffer from anxiety. Despite threats and unfavorable environmental conditions, resilient individuals can successfully adapt themselves to circumstances [44, 45]. Therefore, it can be inferred that resilient individuals’ self-efficacy decreases less seriously in encountering with unexpected events.

According to Bandura, emotional and physiologic states such as anxiety, stress and tiredness are sources conveying self-efficacy beliefs to individuals [19, 40]. The perception of individuals regarding physiologic and emotional responses to a specific behavior is another source of self-efficacy, since these perceptions may affect their judgments about their abilities. Similar to a previous study conducted on medical students in the United Stated [46], the present study showed that negative emotions like anxiety could affect self-efficacy beliefs. Although physiologic and emotional responses did not emerge in the present research as a separate category, the results of this study showed that these physiologic responses could reduce the educators’ self-efficacy as a result of factors such as lack of experience of the educator, encountering unexpected events, inadequate professional knowledge and skills, inability to earn the trust of the clients, negative effect of the educator’s self-concept, and effect of vicarious experiences. Therefore, it can be mentioned that healthcare providers considering an educational situation a stressful event are less able to control that situation, thereby resulting in lower perceived self-efficacy for health education practice.

The forth factor affecting self-efficacy in this study was “vicarious experiences”, which is consistent with the fourth source of self-efficacy according to Bandura [19, 37, 38]. This result is similar to the finding obtained in Usher. It indicated that in addition to interpreting the results of their actions, individuals build their self-efficacy beliefs through vicarious experiences by observing others [39]. Students would evaluate their capabilities in many academic duties, in which they had no experience, by comparing the performances of others. If the majority of the classmates obtain lower marks, the self-efficacy of the student rises, and vice versa [39]. In the present study, health educators create vicarious health education experiences with their colleagues in similar situations, particularly, when they have limited previous experience, they are more sensitive to this issue. As mentioned in the findings section, one of the educators said that as a result of having vicarious experiences, he/she came to believe that he/she would be able to educate others effectively. Additionally, when individuals compare their own situation with others, if they feel they are at a lower level, they feel stressed, and these emotional signs of stress reduce their self-efficacy.

Health educators’ “self-concept” is the fifth factor affecting their perception of self-efficacy in successful educational sessions. For example, if this self-image is not positive, it can lead to anxiety and reduce his/her self-efficacy. The question is “How can self-concept help the health educator, and what benefits its understanding would have?” Self-concept can predict future behaviors. Self-concept is able to form behavioral motivations and direct them toward specific behaviors [47]. The results of this study showed that participants who believed in their feelings, could educate others, based on earning the clients’ trust. In one study conducted by Aghabarary into nurses and patients’ views on barriers to communication, it is reported that the confidence and trust of the patient in the practical and scientific capability of the healthcare staff are the most important factors in this regard [48].

In the present study, the participants stated that if healthcare personnel did not have “professional skills and knowledge”, they would become anxious; this feeling negatively affects their self-efficacy. According to the study by Haghbaghery and Salsali, self-efficacy originates from personal characteristics, but the level of knowledge and work-related social relationships affects individuals’ professional self-efficacy. In this study, the participants mention factors such as the use of inappropriate methods of education, casting doubt on the scientific and technical competence of the healthcare personnel, thereby affecting their self-efficacy [36]. In the present study, the participants mentioned lack of up-to-date information as one of the factors reducing self-efficacy. Studies suggest that the majority of the healthcare personnel feel they do not have the self-confidence regarding the skills required for educating others. However, clients’ educational needs can motivate them [14, 49, 50].

## Conclusion

Some factors associated with self-efficacy as extracted by the present study are largely in line with the sources proposed by Bandura but in a more extensive sense. Some new categories such as “encountering unexpected events” and “clients’ trust”, which affected the healthcare providers’ professional self-efficacy beliefs in health education practice, emerged from the study. According to the results of our study, it seems that it is necessary to develop educational programs based on the extracted factors to increase health educators’ self-efficacy.

### Strengths and weaknesses

One of the strengths of the study was participation of people from diverse backgrounds in the study, for example from both health and treatment fields. In addition, the emergence of the concept of sexuality as affecting the self-efficacy of health educators in the Iranian culture was among the strengths of the present study. Furthermore, the results of this research can be used to provide more precise instruments to measure these factors or design empowerment interventions.

In the present study, although the nature and quiddity of the self-efficacy sources were clearly explained and comparisons and contrasts with Bandura’s sources were made, no findings were offered about how these sources were affected, particularly in cases where this leads to reduced self-efficacy in health education. Moreover, no findings were presented about how these sources could be measured and what their relationship with self-efficacy was. Thus, it is suggested that further studies devise appropriate instruments to measure the self-efficacy sources in health education and subject the devise instruments to psychometric methods so that the effects of different interventions on the improvement of self-efficacy in health education could be studied.

## Abbreviation

SE: Self-efficacy
